# A novel inductively coupled capacitor wireless sensor system for rapid antibiotic susceptibility testing

**DOI:** 10.1186/s13036-023-00373-5

**Published:** 2023-08-18

**Authors:** Yikang Xu, Dacheng Ren

**Affiliations:** 1 Department of Biomedical and Chemical Engineering, Syracuse University, Syracuse, NY 13244 USA; 2 BioInspired Institute, Syracuse University, Syracuse, NY 13244 USA; 3 Department of Biology, Syracuse University, Syracuse, NY 13244 USA; 4 Civil and Environmental Engineering, Syracuse University, Syracuse, NY 13244 USA; 5 Present address: Department of Biomedical and Chemical Engineering, College of Engineering & Computer Science, Syracuse University, 223K Link Hall, Syracuse, NY USA

**Keywords:** Biosensor, Antimicrobial susceptibility test, Permittivity, Antibiotic resistance

## Abstract

**Background:**

The increasing prevalence and severity of antimicrobial resistance (AMR) present a major challenge to our healthcare system. Rapid detection of AMR is essential for lifesaving under emergent conditions such as sepsis. The current gold standard phenotypic antibiotic susceptibility testing (AST) takes more than a day to obtain results. Genotypic ASTs are faster (hours) in detecting the presence of resistance genes but require specific probes/knowledge of each AMR gene and do not provide specific information at the phenotype level. To address this unmet challenge, we developed a new rapid phenotypic AST.

**Result:**

We designed a new electrochemical biosensor based on the concept of magnetically coupled LC sensors. The engineered LC sensors can be placed in 96-well plates and communicate the reading remotely with a receiver coil for signal analysis. The sensors were validated by monitoring the growth of *Escherichia coli*, *Staphylococcus aureus*, and *Pseudomonas aeruginosa* in the presence and absence of different antibiotics. Drug-resistant strains were used as controls. Bacterial growth was detected within 30 min after inoculation, allowing rapid determination of antibiotic susceptibility at the phenotype level. The sensor also functions in the presence of host proteins when tested with 2% FBS in growth media.

**Conclusions:**

With the compatibility with 96-well plates, this label-free rapid 30-min AST has the potential for low-cost applications with simple integration into the existing workflow in clinical settings.

**Supplementary Information:**

The online version contains supplementary material available at 10.1186/s13036-023-00373-5.

## Introduction

Drug-resistant infections present great challenges, especially in hospital settings. About 30% of ICU patients are affected even in high-income countries; and the number is two to three folds higher in low- and mid-income countries [1]. According to a recent WHO report, 2.8 million AMR infections occur each year in the U.S. alone [2]; and at least 1.27 million people died from AMR infection in 2019 worldwide [3]. It is predicted that, by 2050, there could be 10 million deaths per year globally if no effective treatment for AMR is available [4]. Rapid detection of AMR has been proven critical for saving sepsis patients. In an animal study that mimics sepsis, it was revealed that antibiotic treatment administered 12 h after bacterial inoculation resulted in a 96-h survival rate of 80%, whereas treatment administered at 15 h had the survival rate sharply decreased to only 13.3% [5]. Thus, rapid ASTs are urgently needed to reduce sepsis mortality and help with antibiotic stewardship programs.

Current methods of pathogen detection by sample cultures have a median growth time of around 13 h [6], which then yields a microbial culture with 10^7^ – 10^8^ CFU/mL for further analysis of antibiotic susceptibility [7]. Without rapid test of antibiotic susceptibility, the patients are often given general antibiotics [8]. This can cause the precious window to prevent patient mortality to be missed by ineffective treatments due to AMR. Traditional phenotypic methods such as dilution methods, agar disk diffusion testing, and gradient diffusion methods, typically take 1–2 days to generate reliable results [9, 10]. Genotypic ASTs, on the other hand, directly detect biomarkers associated with resistance using molecular detection tools such as qPCR, whole-genome sequencing, and MALDI-TOF. Such tools are highly sensitive and could produce a report in hours [11]. However, genomic approaches require detailed knowledge of AMR gene sequences in advance, and thus cannot detect newly developed resistance mechanisms [9]. There are other new approaches being developed such as optical imaging to identify bacteria and/or track bacterial growth in microfluidic devices [9, 12–16], pH sensors for tracking byproducts of bacterial growth [17], bioluminescence assay of ATP [18], magnetic sensors with antibody coating [19], and electrochemical biosensors with peptide or antibody coating [20, 21], etc. These novel approaches provide high sensitivity and specificity, but require extensive image/data processing, complex sample preparation, and/or sophisticated equipment that could be challenging to operate in clinical settings. Additionally, ASTs based on single-cell detection/monitoring have challenges in polymicrobial infections with mixed microbial populations [22]. Thus, we are motivated to develop a rapid high throughput phenotypic AST that is readily scalable, can detect antimicrobial susceptibility at the population level, and can be easily integrated into clinical settings.

Here we report our design based on a new LC sensor. The application of LC sensors in medical devices dates back to 1960s [23]. The magnetic coupling between the sensor coil and the detection coil transmits AC electricity to the sensor circuit where the resonant frequency of the inductor (L) and capacitor (C) can be computed based on the frequency spectrum. The resonant frequency changes in response to the surrounding environment, allowing the detection of substances that change the surface property of the sensor. The wireless nature of the sensor enables sensing in hard-to-access locations either in an instrument or the human body such as wound health sensing [24]. In addition, LC sensor requires no integrated power to operate. Thus, it is possible to make small form factor LC sensors with a long life span. These advantages make LC sensors attractive in designing biomedical devices where the sensors are often sealed and require low to no maintenance to operate in biological environments. However, this technology did not come onto the main stage of remote query systems until the development of microelectromechanical systems (MEMS), which enable small-scale LC sensors to be embedded into medical devices and microfluidic devices. Among the applications of this technology [25], a LC resonant sensor for the passive monitoring of bacterial growth in a large volume of agitated medium was developed. But protein adsorption over time was not addressed and the method had a rather long detection time of 8 h. Impedance measurements have also been shown to achieve high sensitivity in microbial detection, e.g., 30 CFU [26]. However, the current system requires rather complex coating and is not designed for AST. To address the unmet challenges in AST, we developed a new LC sensor based on rational design. It significantly reduces the response time to 30 min and is functional in the presence of host proteins.

**Fig. 1:**
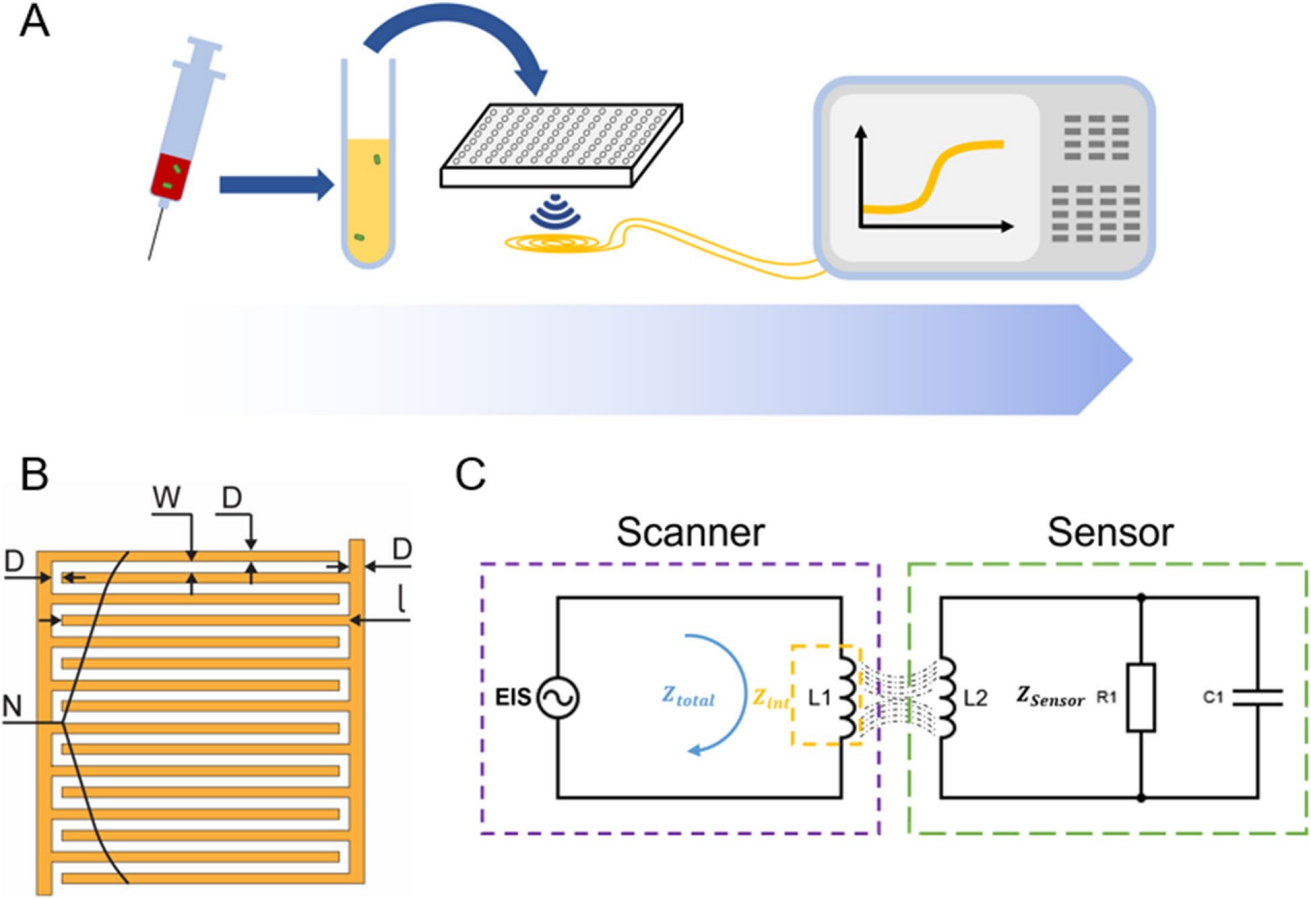
**A** Schematic of AST using inductively coupled communication between the LC sensor and detection coil connected to EIS. The diluted bacterial culture with desired antimicrobial added is aliquoted into individual wells on a 96-well plate. The sensor wrapped around the internal walls of the well plate communicates wirelessly with an EIS analyzer to capture bacterial growth. **B** An illustration of the IDC and its parameters, where N is the number of digits in total, ɭ is the length of each digit, D is the gap between digit, and W is the width of the digits. **C** An illustration of the resonant circuit design, where the inductors represent the coils and the parallel resistor R1 and capacitor C1 represent the IDC on the sensor side, with Z_sensor_ representing the total impedance of the coil and the IDC. On the scanner side, Z_int_ is the intrinsic impedance of the coil, Z_total_ is the total measured impedance of the coil. Also included is a signal generator/analyzer (EIS)

This new rapid phenotypic AST method was developed based on wirelessly magnetically coupled LC sensors, that can exploit the capacitive nature of bacteria [27]. It enables bacterial growth monitoring and antibiotic susceptibility testing without the need for sample enrichment, and species-specific surface modifications or coatings (Fig. 1A). This sensor is expected to be low-cost and requires no special sample handling. It can be integrated into 96-well plates to achieve AST within 30 min after a positive culture is identified.

## Methods and materials

### Bacterial media, reagents and materials

Low salt LB medium contained 0.5 g/L NaCl, 10 g/L tryptone, and 5 g/L yeast extract, supplemented with 1–5% fetal bovine serum. Antibiotics tested in this study include ampicillin, ofloxacin, ciprofloxacin, vancomycin, and tobramycin. The sensors and receiver plate were fabricated on a flexible polyimide flex circuit board (Custom ordered from PCBWay Prototype to volume production Factories, Shenzhen, China). Oil-based polyurethane protective spray coating was purchased from MINWAX (New York, NY). Permittivity data were measured using an EIS analyzer (Model E4990A-20, Keysight, Santa Rosa, CA).

### Sample preparation

Overnight bacterial cultures were grown in 25 mL low salt LB medium at 37 °C for 16 h with shaking at 200 rpm. To test antibiotic susceptibility, overnight cultures were used to inoculate low salt LB medium with a starting OD_600_ of 0.001. Three hundred µL of the inoculant was then aliquoted to a 96-well plate with LC sensor inserts and quickly transferred to a 30 °C culture room for growth monitoring.

### Broth microdilution AST

A 96-well plate was inoculated with 200 µL of low salt LB medium with designated antibiotics at different concentrations and bacteria with a starting OD_600_ of 0.0001. The inoculated microplates were transferred to a 30 °C culture room for 16 h of incubation and then visually inspected to determine MIC.

### Permittivity calculation

In order to calculate the complex permittivity of a bacterial culture, an equation can be derived from parameters of the LC sensor and the resonant frequencies collected from the EIS [28–30]:

1 $$\varepsilon =\frac{{C}_{1}}{k{\varepsilon }_{0}}-{\varepsilon }_{sub}+\frac{1}{k{R}_{1}{\omega }_{zero-inductance}{\varepsilon }_{0}}$$

In which ${\varepsilon }_{0}$ is the permittivity of free space, ${\varepsilon }_{sub}$ is the permittivity of substrate material, ${\omega }_{zero-inductance}$ is the zero-inductance frequency of the system, and C1, R1 are components of the sensor circuit (Fig. 1C). Additionally, $k$ is the cell constant of the IDC defined by:

2 $$k=\frac{\text{l}\left(N-1\right)K{\left[1-{\left(\frac{D}{D+W}\right)}^{2}\right]}^\frac{1}{2}}{\left.2K[\frac{D}{D+W}\right]}$$

All parameters needed to calculate k can be found based on the sensor design (Fig. 1B), except for the elliptic integral of the first kind expressed as K[A]. Using Eqs. 1 & 2, a permittivity vs time plot was generated. Thus, the permittivity changes in the system can be tracked to monitor bacterial growth, allowing rapid assay of antibiotic susceptibility.

### Signal processing

The resonant circuit consists of two sides, including a circuit with a coil and an IDC on the sensor side, and a detection coil and a signal generator/analyzer (Fig. 1C) on the scanner side. Circuit analysis [31] could be used to obtain the following equation from this circuit:

3 $${Z}_{total}={Z}_{int}+\frac{{\omega }^{2}{M}^{2}}{{Z}_{sensor}}$$

In this equation, Z_int_ is considered as the background impedance of the system and is subtracted using the built-in function of the EIS. Z_sensor_ can be represented using the frequency domain as:

4 $${Z}_{sensor}=j\omega {L}_{2}+\frac{{R}_{1}}{1+j\omega {R}_{1}{C}_{1}}$$

Substituting Eq. (4) into Eq. (3), and combining with the subtraction of background noise ${Z}_{int}$ mentioned above, the representation of total impedance on the scanner side becomes:

5 $${Z}_{total}=\frac{{\omega }^{2}{M}^{2}{R}_{1}}{{\left({R}_{1}-{\omega }^{2}{R}_{1}{C}_{1}{L}_{2}\right)}^{2}+{\omega }^{2}{{L}_{2}}^{2}}+j\frac{\left.{\omega }^{2}{M}^{2}(\omega {R}_{1}{C}_{1}\left({R}_{1}-{\omega }^{2}{R}_{1}{C}_{1}{L}_{2}\right)-\omega {L}_{2}\right)}{{\left({R}_{1}-{\omega }^{2}{R}_{1}{C}_{1}{L}_{2}\right)}^{2}+{\omega }^{2}{{L}_{2}}^{2}}$$

It is important to note that in this equation, ω (frequency), M (mutual inductance), and L2 (inductance of sensor coil) are all known parameters that can be controlled either by changing the input or the design of the IDC. This means only two parameters, the R1 and C1, are unknown and can be solved using Eq. 5 after setting the imaginary part of the impedance to 0 at the zero-reactance frequency (Eq. 6) and taking the derivative of the real part of the Eq. (3) to set ω to resonant frequency (Eq. 7).

6 $${\omega }_{zero-inductance}=\sqrt{\frac{1}{LC}-\frac{1}{{R}^{2}{C}^{2}}}$$

7 $${\omega }_{resonant}=\sqrt{\frac{1}{LC}}$$

With these two frequencies calculated, the complex permittivity of the IDC can be calculated with the two equations mentioned above (Eqs. 1 & 2).

### Electromagnetic coupling analysis of the sensor

**Fig. 2:**
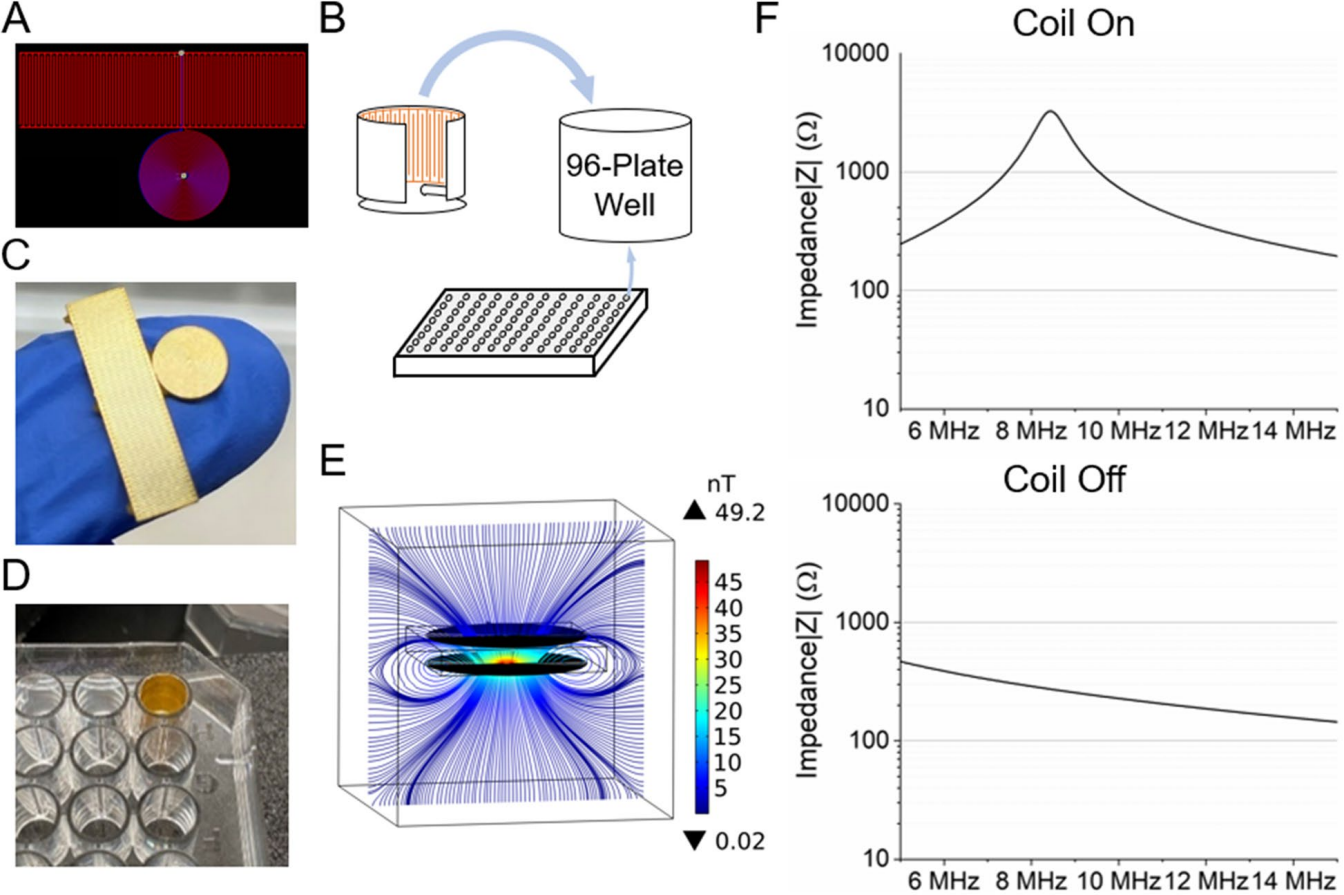
Implementation of the LC sensor system in 96 well-plate. **A** Design of a single sensor. **B** The orientation of the folded sensor fitting in a single well of a 96 well-plate. **C** Picture of a fabricated sensor before folding. **D** Picture of a 96 well-plate well with a sensor folded and inserted. **E** COMSOL simulation of magnetic coupling between the receiver coil and the sensor coil across a piece of polystyrene plastic, showing the 96 well plate bottom is magnetically transparent and will not prevent the coils from coupling. **F** The frequency sweep data before and after the sensor is coupled to the receiver coil. The line represents the absolute impedance $\left|Z\right|$ of the system

Two identical coils both with 0.035 mm wire thickness, 0.06 mm wire width, 0.06 mm wire gap, 50 turns with 25 turns on each side of the polyimide flex PCB was brought together within 1.2 mm distance separated by a 0.9 mm thick polystyrene plastic well bottom of a standard 96-well plate. Power was supplied to the receiver coil by the impedance EIS at 50 µA current level with frequencies ranging from 1 MHz to 12 MHz. Figure 2E shows the COMSOL simulation of the magnetic coupling of the two coils. The frequency sweep was performed with the EIS at a resolution of 1600 points, and the absolute impedance and phase shift of the system were recorded before and after the sensor was brought within the coupling range of the two coils (Fig. 2F). The result shows that the polystyrene 96-well plate is magnetically transparent enough to have a negligible effect on the coupling of the two coils.

### Construction of the sensor system

Bacterial cultures were diluted and aliquoted into a standard 96-well plate with a sensor inserted in each well. A receiver coil connected to a Keysight E4990A-20 EIS on the bottom of the 96 well plates wirelessly communicates with the sensor and scans a spectrum of electrical wavelength to identify the resonance frequency between the sensor and the receiver coil. The resonance frequency of the system was recorded every 5 min, combined with the parameter of the sensor to calculate the permittivity of the bacterial culture. The permittivity readout is plotted as a time series, and the slope of the curve over the initial 30 min was used to access the sensitivity score of bacteria to each tested antibiotic. The baseline of the sensitivity score is determined by acquiring the time sequence slope in sterile medium (for growth detection) or antibiotic-free cultures (for AMR detection).

All experiments were performed with cultures incubated at 30 °C without shaking, and with an inoculation OD_600_ of 0.001 unless noted otherwise. The positive and negative ends of the receiver coil are situated in a diecast aluminum electromagnetically insulated box, which is connected to the EIS via a pair of twisted and insulated stranded copper wires.

The sensor was first mounted onto a double-sided adhesive sheet and had the single-layer protective polyurethane spray coating applied. The entire sheet of coated sensors was then left in a desiccator for 48 h to ensure complete evaporation of solvent and curing of the coating material. Individual sensors were then removed from the sheet with adhesive backing, rolled up as cup sleeves and placed inside of the wells. To avoid interference from protein and bacteria settling effect in a static culture environment, the sensing component of the sensor is placed vertically to the bottom, lining the wall of the well. Finally, the entire 96-well plate was placed in a UV Clave ultraviolet sterilization chamber for a one-hour sterilization cycle. Three sensors were randomly selected to establish a baseline for each batch of sensors to verify successful coating. On the EIS platform, a MATLAB program was used to trigger the equipment every 5 min for a 35 min duration, the program then took the readout, searched for the resonance frequency, and saved it as time series for further analysis. The file generated by the MATLAB program is written in VBScript to interface with the EIS to perform a preset 1600 points sweep within a 1 MHz range near the initial resonance frequency. The experimental setup is shown in Fig. 2B.

## Results and discussion

### Sensor design

**Fig. 3:**
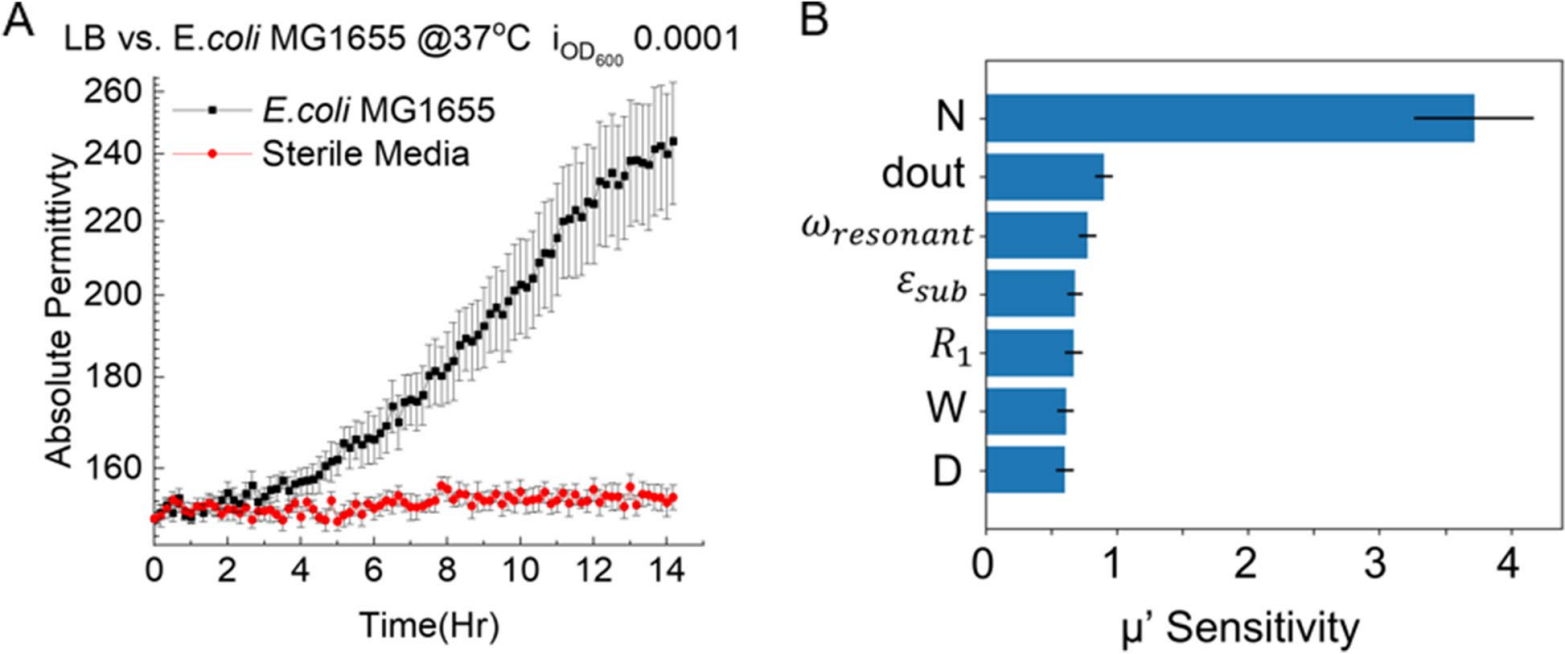
**A** Overnight growth curve of *E. coli* MG1655 ASV in LB medium obtained with initial sensor design. The culture started with an inoculation OD_600_ of 0.0001. **B** Method of Morris multivariable sensitivity analysis of Eq. 5 where the newly introduced term d_out_ is the outer diameter of the sensor

Our initial iteration of the sensor design was composed of a fiberglass printed circuit board with 5 turns of coil on both sides of the PCB with 11 0.5 mm digits spaced 0.5 mm apart. The sensor was placed inside of an upside-down 50 mL conical tube with 20 mL of LB inoculated with *E. coli* MG1655 ASV at OD_600_ of 0.0001. The assembly was then placed on a shaker set to 200 rpm overnight in a 37 °C culture room. The growth curve obtained from the system is shown in Fig. 3A. The sterile medium did not show a significant change in the signal over time. As expected, the growth curve displays lag phase, exponential phase, and transition to stationary phase. However, the slower detection speed of the sensor was unsatisfactory, which motivated a redesign of the sensor aiming to achieve a faster detection speed.

After performing an analysis of the equation with the method of Morris sensitivity analysis in SALib (Sensitivity Analysis Library in Python) [32], it was revealed that the number of turns in the coil had the largest influence on the permittivity value, followed by the outer diameter of the coil and the distance D between digits (Fig. 3B). This was taken into consideration when redesigning the sensors to achieve the highest sensitivity. After several iterations of sensor design including changing the substrate of the sensor from a thick resin-reinforced board to a thin polyimide film, and adopting a round shape coil, the final design of the sensor with high coil turn counts and high quantity IDC digits showed rapid detection capability with high sensitivity. The wire dimensions were intentionally kept large enough for conventional circuit printing techniques enabling easy sensor fabrication with existing mature manufacturing processes.

### Bacterial growth monitoring and AST

**Fig. 4:**
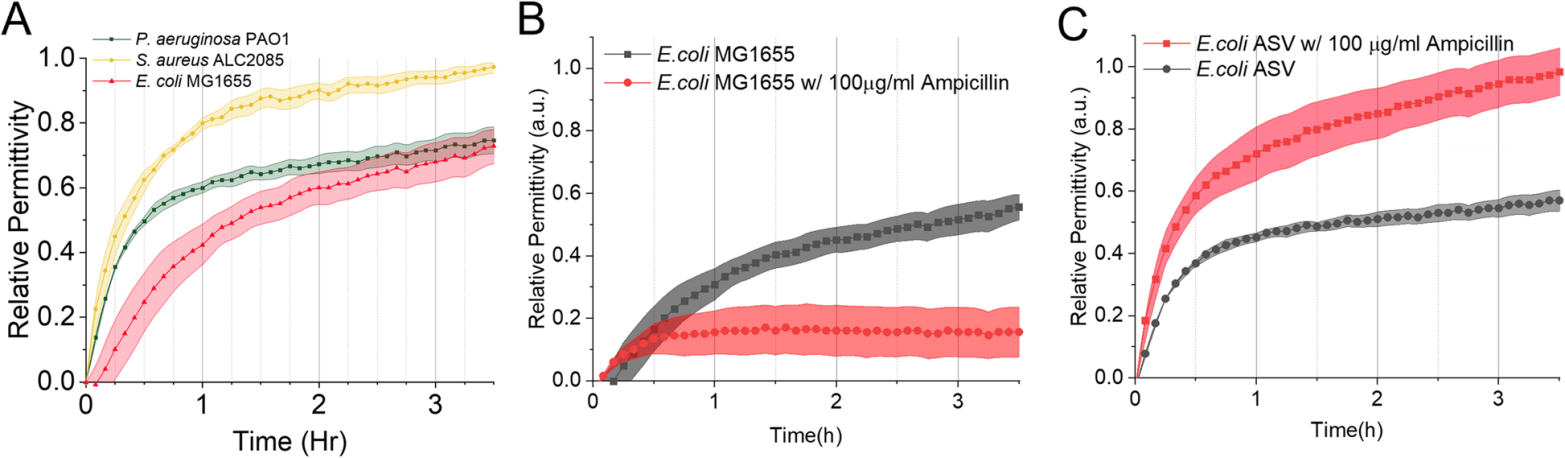
Monitoring bacterial growth using the new sensor system. The permittivity values are normalized to 0–1 scale with *S. aureus* curve final value. The permittivity values in figure (**B **& **C)** are normalized to 0–1 scale with *E.coli* ASV curve final value. **A** Growth curve of *E. coli* MG1655, *S. aureus* ALC2085, and *P. aeruginosa* PAO1 in the sensor system over 3.5 h with permittivity measured at 5-min intervals. **B** Growth of *E. coli* MG1655 (wild-type) with and without 100 µg/mL ampicillin treatment. **C** Growth of *E. coli* MG1655 ASV (ampicillin resistant strain) with and without ampicillin treatment

To ensure the conductive nature of the media doesn’t interfere with the sensor, low salt LB was selected as the medium for bacterial growth in this study. The high resistance introduced by low salt LB provides a lower background level making the resonant frequency shift in the system more detectable. Baseline readout of the sterile medium was established and subsequently subtracted as background from the experimental data below. Initial growth monitoring was tested with *E. coli* MG1655 (Fig. 4A). Data revealed that compared to the lag phase observed in the growth curve based on OD_600_, relative permittivity was more sensitive in detecting bacterial growth. Upon inoculation, there was an immediate increase in permittivity, likely due to nonspecific adsorption of proteins in the growth medium onto the polyurethane coating. Similar phenomena were observed when measuring the baseline, where there was an initial moderate increase in signal for the sterile medium alone. Further increase due to specific bacterial growth (after subtracting the signal from the sterile medium) was recorded within 30 min after inoculation, much earlier than OD_600_ measurement (around 5 h). The growth curve collected by the system showed a rapid increase in relative permittivity which then slowed down over time and eventually reached a plateau. The presence of a plateau is presumably due to the saturation of free space on the sensor surface by bacterial growth and associated byproducts. Further experiments were done on two more different strains of both Gram-positive and Gram-negative bacteria to test if the functionality of the sensor extends beyond just *E. coli*. As shown in Fig. 4A, not only do all three strains elicit similar responses over the 3.5 h monitoring period, the three strains showed some segments of different patterns of growth, which can be investigated in future research for potential strain identification using specific media.

To determine if this sensor can be used for AST, an ampicillin sensitive strain *E. coli* MG1655 and ampicillin resistant strain *E. coli* MG1655 ASV were compared for growth in the absence and presence of ampicillin. Both strains were dosed with 100 µg/mL ampicillin at the time of inoculation and cultured for 4 h, which is known to induce cell lysis in sensitive *E. coli* strains [33]. As shown in Fig. 4B & C, *E. coli* ASV strain grew in the presence of ampicillin while the wild-type *E. coli* MG1655 strain was inhibited by the ampicillin treatment as expected, all of which was observed within 30 min. The low initial increase in relative permittivity may be from bacteria in the suspension being lysed, and the cellular content increased the permittivity near the sensor surface.

**Fig. 5:**
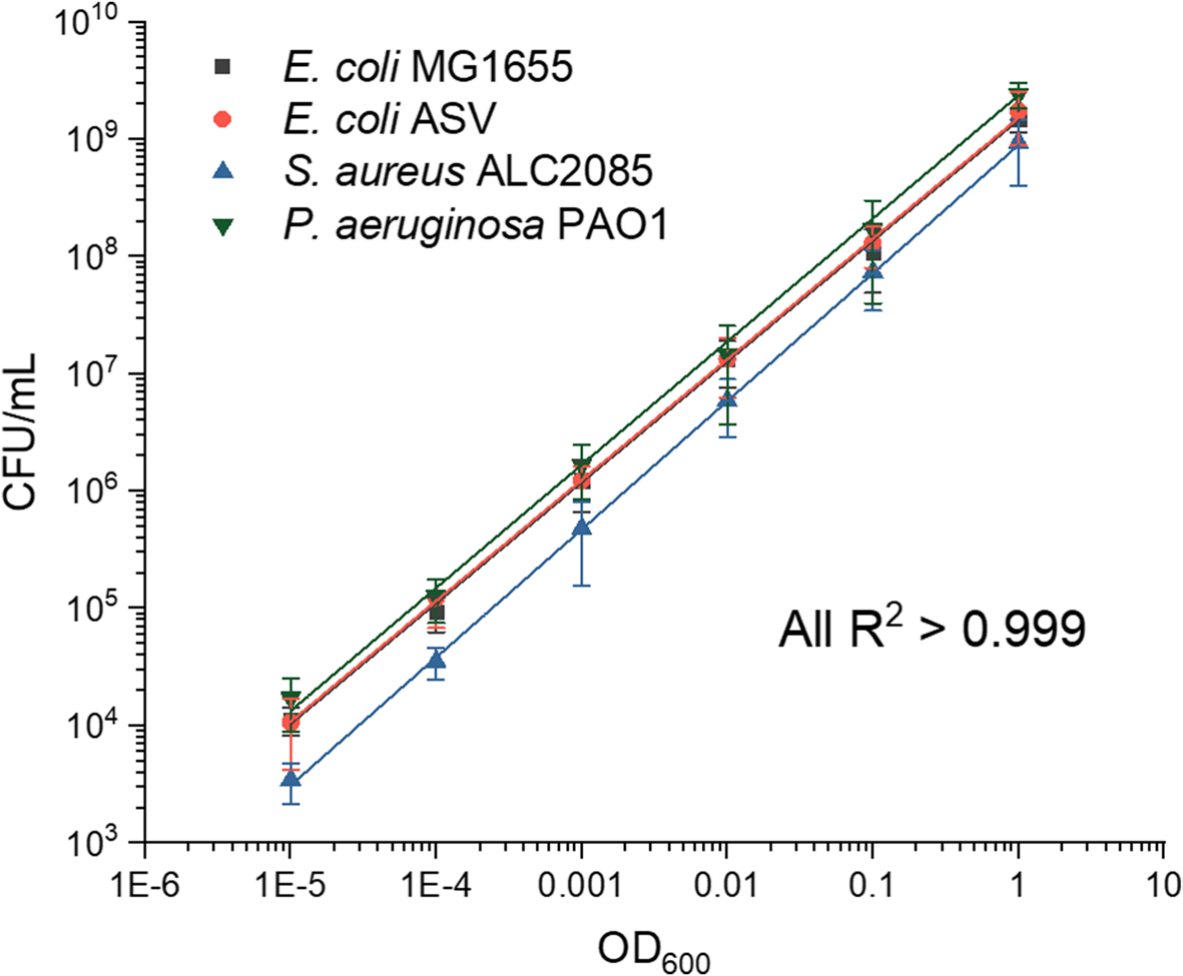
Linear correlation between OD_600_ and CFU for cultures of *E. coli* MG1655, *E. coli* MG1655 ASV, *S. aureus* ALC2085, and *P. aeruginosa* PAO1 (*R*^2^ > 0.999 for all)

To further corroborate the results and validate that OD_600_ measurements accurately represent bacterial growth, we counted the CFU of the strains involved in this study. The OD_600_ and CFU/mL data show clear linear correlation, indicating that OD_600_ is a valid measurement for monitoring growth and comparing with sensor data (Fig. 5).

Subsequent experiments were performed on more antibiotics and all three previously tested bacterial species. In order to develop a standard method, the test time was set universally as 30 min with 5-min intervals of sampling, since all tested growth curves showed initial signs of resistance or growth in that time frame (Fig. 4C).

**Fig. 6:**
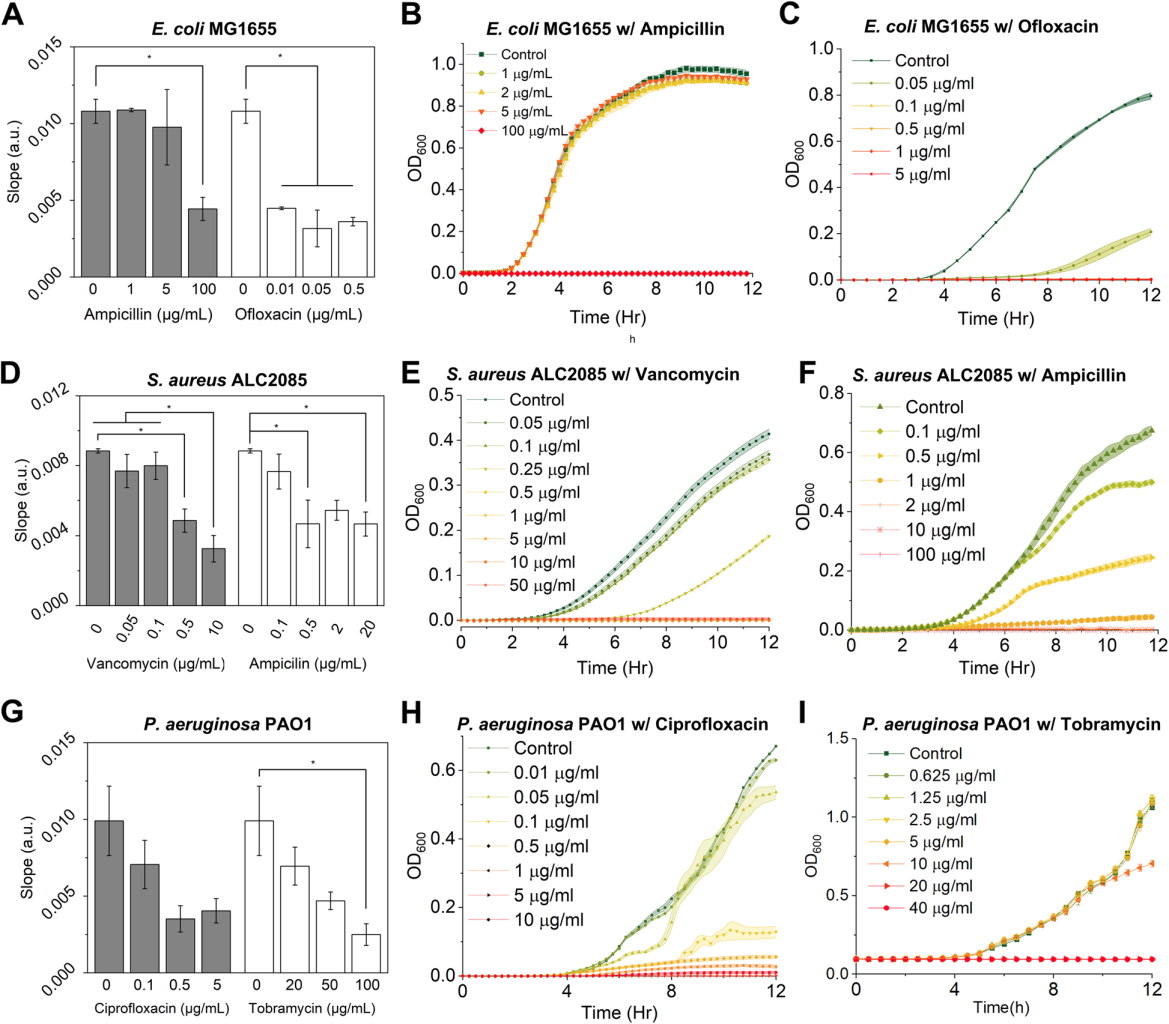
AST results based on the slopes of permittivity curves (**A**, **D**, **G**) and growth curves based on OD_600_ (**B**, **C**, **E**, **F**, **H**, **I**). **A** Slopes of permittivity curves of *E. coli* MG1655 with and without ampicillin and ofloxacin. **B** OD_600_ of *E. coli* MG1655 treated with different concentrations of ampicillin. **C** OD_600_ of *E. coli* MG1655 treated with different concentrations of ofloxacin. **D** Slopes of permittivity curves of *S. aureus* ALC2085 with and without vancomycin and ampicillin. **E** OD_600_ of *S. aureus* ALC2085 treated with different concentrations of vancomycin. **F** OD_600_ of *S. aureus* ALC2085 treated with different concentrations of ampicillin. **G** Slopes of permittivity curves of *P. aeruginosa* PAO1 with and without ciprofloxacin and tobramycin. **H** OD_600_ of *P. aeruginosa* PAO1 treated with different concentrations of ciprofloxacin. **I** OD_600_ of *P. aeruginosa* PAO1 treated with different concentrations of tobramycin

At the same time, to obtain more rapid detection of growth and distinguish resistant vs. sensitive strains, a linear regression was opted in on the relative permittivity. The slope of the best-fit line was then used to determine the sensitivity of the tested strain toward an antibiotic. It can be seen in Fig. 6 that the dose dependence of bacterial susceptibility to an antibiotic can be seen in both LC sensor system and OD_600_ measurement, where trends of growth inhibition after 6–10 h in OD_600_ measurement were captured by the slope of permittivity measurement substantially earlier in just 30 min.

To further validate this assay, a microdilution MIC test was performed with the same bacterial strains and antibiotics included in this study, while more concentrations were included in this microdilution test to determine the exact MIC. Based on the microdilution test, the MICs were found as follows: *E. coli* MG1655 (ampicillin: 12.8 µg/mL; ofloxacin: 0.016 µg/mL), *S. aureus* (vancomycin: 0.2 µg/mL; ampicillin: 0.8 µg/mL), *P. aeruginosa* PAO1 (ciprofloxacin: 0.256 µg/mL; tobramycin: 51.2 µg/mL). These results are consistent with the senor data in Fig. 6.

### Performance in the presence of host proteins

**Fig. 7:**
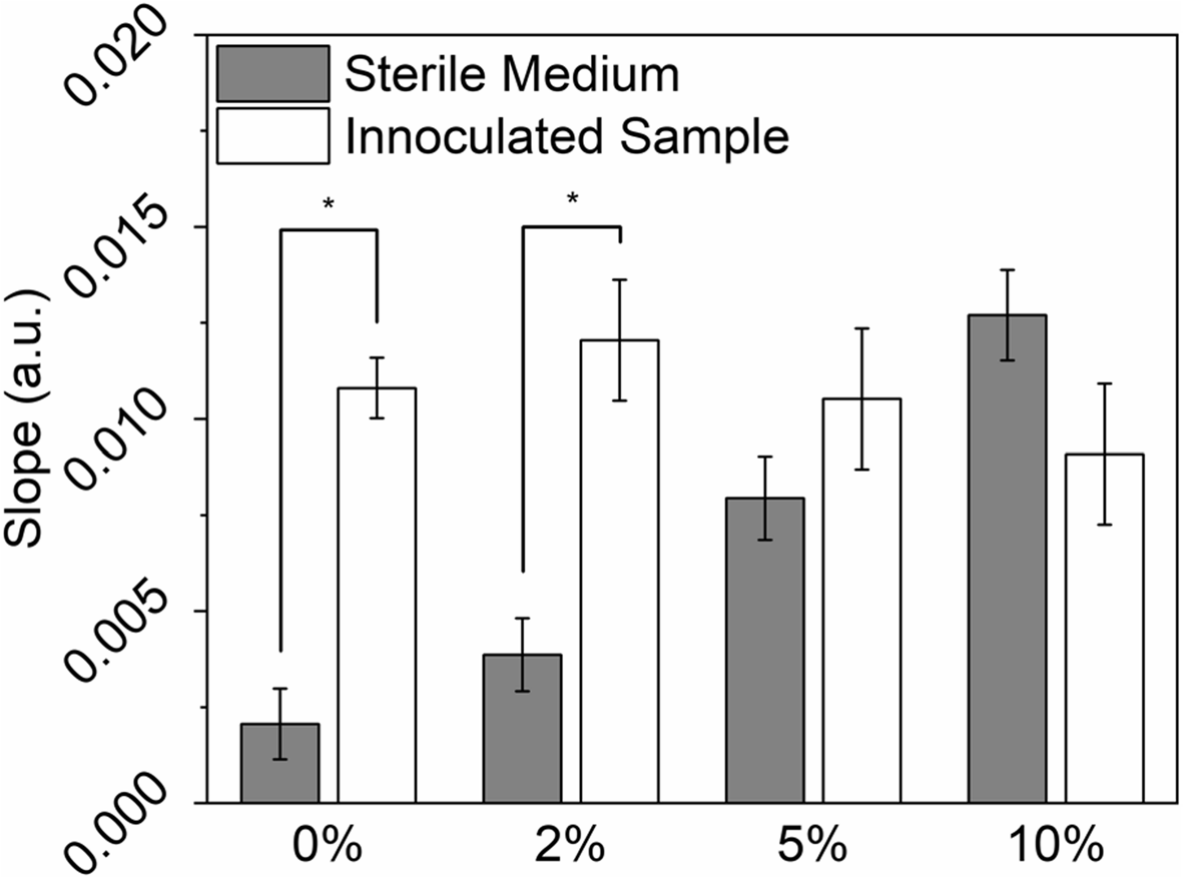
Slopes of permittivity curves of *E. coli* MG1655 cultured in low salt LB supplemented with different concentrations of FBS

Samples collected in a clinical setting are often complex. Whole blood, plasma, serum, urine, or other liquid from the human body contains significantly more types and larger amounts of proteins and cells compared to laboratory culture media. Thus, it is necessary to test the sensor system with the addition of complex components in order to evaluate the feasibility of implementing this system in a clinical setting where sample preparation beyond dilution and centrifugation is seldom performed. For the above reasons, fetal bovine serum was selected to mimic host serum and added to low salt LB at different percentages. The response of the sensor system is shown in Fig. 7. Encouragingly, at lower concentrations, the sensor functioned well for sensing bacterial growth suggesting that there is sufficient free space near the sensor surface. For low salt LB dosed with 2% of FBS, the growth of *E. coli* wild-type strain MG1655 was captured by the LC sensor system with little interference from the serum. This demonstrates the possibility of using these sensors in clinical settings with simple dilution. When the amount of FBS reached 5%, the response was lost, likely because the free space thickness was saturated by protein adsorption. Based on these results, we expect that the system will function with clinical samples because the seeding cells are from cultured samples with host proteins significantly diluted. Additional testing with clinical samples is needed to further validate this system and it is part of our further work.

The coating material and coating thickness of the chips can also be optimized to achieve higher accuracy and resistance to fouling from the growth media or cellular products. A better optimized coating could also further decrease detection limit and broaden detection range. The results of 2% FBS demonstrate the possibility to test clinical samples after simple dilution. To handle even higher amounts of serum if needed, the IDE design could be adjusted to increase the saturation thickness the upper concentration limit of FBS content. The saturation thickness can be described by Eqs. 8, 9 and 10, where a1 and a2 are both functions of digit width W and gap width D [34].

8 $${d}_{sat}=-\frac{D}{{a}_{1}}\mathrm{ln}\left(\frac{0.005}{{a}_{2}}\right)$$

9 $${a}_{1}=114.97{(W+D)}^{3}+28.75{(W+D)}^{2}-9.183\left(W+D\right)+1.631$$

10 $${a}_{2}=1293.21{(W+D)}^{3}+164.87{(W+D)}^{2}-6.521\left(W+D\right)+6.105$$

With thicker free space for sensing, the system can be further optimized to capture more complex samples and longer-term growth patterns. As a part of our future work, the sensor would also be challenged with actual clinical samples to further test the tolerance of the sensor surface to fouling and set the thresholds of slopes for AMR detection.

## Conclusions

This study demonstrated the feasibility of rapid AST using LC sensor that is compatible with 96 well plate setting. To our best knowledge, this is the fastest AST test at the phenotypic level to date without using complex equipment. Compared to genotypic ASTs, this system only requires simple sample preparation (dilution only) and can be fitted into an automated workflow for high-throughput detection. The system can generate reports with little computational power without the need for advanced data analysis or the capability of handling massive data sets (e.g., MALDI-TOF). The system is also not limited to the use of specific cartridges for a limited selection of strains. Instead, it resembles the traditional MIC approach where prior knowledge regarding the identity of the species is not required rather the growth inhibition at different dosages alone could determine the antibiotic susceptibility, including polymicrobial infections. But it has a much faster readout (in 30 min). The system can be further optimized in the future by employing high-throughput apparatus to move the plate and scan the wells. This will enable the whole plate to be processed in a relatively short amount of time, and is part of our ongoing work.

### Supplementary Information

**Additional file 1.**

## Data Availability

The dataset(s) supporting the conclusions of this article is(are) available upon request. Please contact the corresponding author for data requests.

## Abbreviations

AMR: Antimicrobial resistance
AST: Antibiotic susceptibility test
OD: Optical density
LB: Lysogeny broth
FBS: Fetal bovine serum
HAI: Healthcare-associated infections
ICU: Intensive care units
MALDI-TOF: Matrix-assisted laser desorption/ionization coupled to time-of-flight mass spectrometry
MEMS: Microelectromechanical systems
PCB: Printed circuit board
IDC: Interdigitated capacitor
EIS: Electrochemical impedance spectroscopy
WHO: World health organization
