# Supplementary figures and images for: A novel inductively coupled capacitor wireless sensor system for rapid antibiotic susceptibility testing
Source: J Biol Eng. 2023 Aug 18;17:54. doi: 10.1186/s13036-023-00373-5 (PMC10439655; doi:10.1186/s13036-023-00373-5)

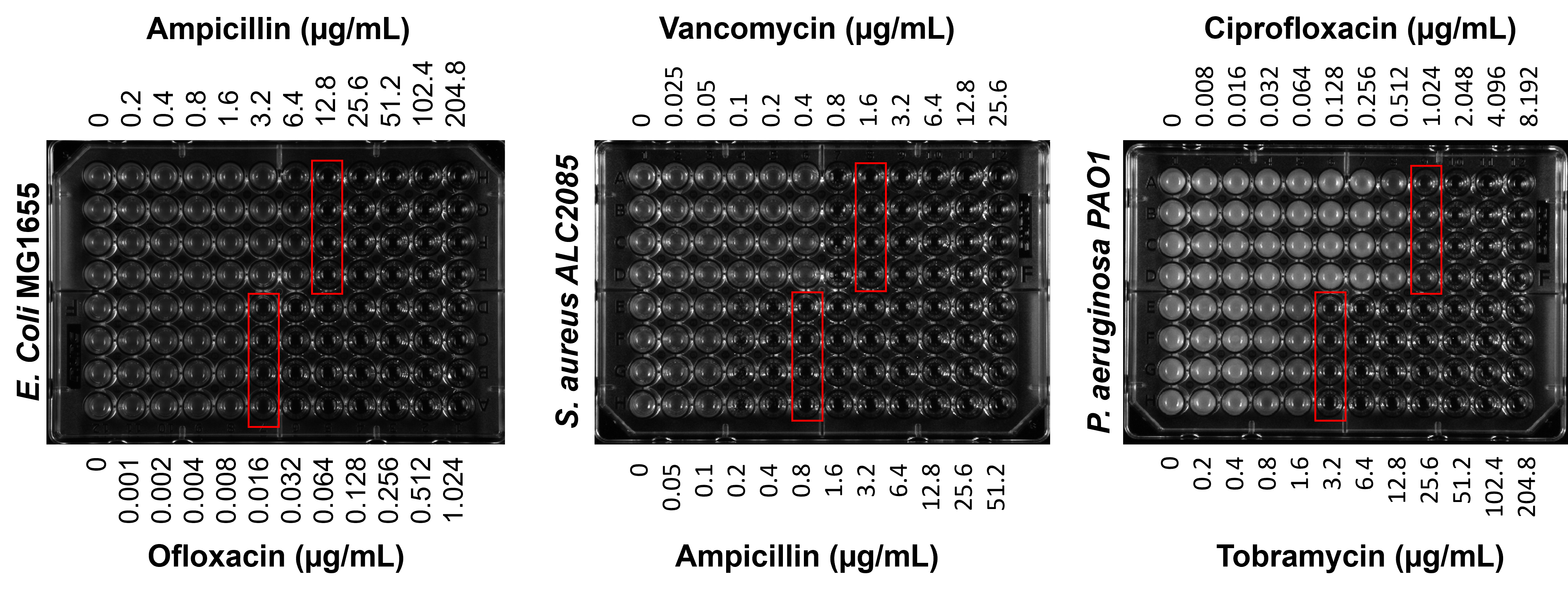

Supplement: Supplementary file 1 — Additional file 1. [file 13036_2023_373_MOESM1_ESM.png]
